# Different Mortality Risks of Long-Term Exposure to Particulate Matter across Different Cancer Sites

**DOI:** 10.3390/ijerph19063180

**Published:** 2022-03-08

**Authors:** Miyoun Shin, Ok-Jin Kim, Seongwoo Yang, Seung-Ah Choe, Sun-Young Kim

**Affiliations:** 1 Department of Cancer Control and Population Health, Graduate School of Cancer Science and Policy, National Cancer Center, Goyang 10408, Korea; 1706104@ncc.re.kr; 2 Environmental Health Research Division, Environment Health Research Department, National Institute of Environment Research, Incheon 22689, Korea; ojkim21@korea.kr; 3 Department of Digital Health, Samsung Advanced Institute for Health Sciences & Technology, Sungkyunkwan University, Seoul 06351, Korea; yangsw@g.skku.edu; 4 Department of Preventive Medicine, College of Medicine, Korea University, Seoul 02841, Korea; seungah@korea.ac.kr

**Keywords:** cancer, cohort, long-term exposure, mortality, particulate matter

## Abstract

Particulate matter (PM) air pollution has challenged the global community and the International Agency for Research on Cancer (IARC) classified airborne particulate matter as carcinogenic to humans. However, while most studies of cancer examined a single cancer type using different cohorts, few studies compared the associations of PM between different cancer types. We aimed to compare the association of long-term exposure to PM (PM_10_ and PM_2.5_) and cancer mortality across 17 different types of cancer using a population-based cohort in the Seoul Metropolitan Area (SMA), South Korea; Our study population includes 87,608 subjects (mean age: 46.58 years) residing in the SMA from the National Health Insurance Services–National Sample cohort (NHIS–NSC) and followed up for 2007–2015. We used the time-dependent Cox proportional hazards model to estimate hazard ratios (HRs) and 95% confidence intervals (95% CIs) of each cancer mortality per 10 μg/m^3^ increase in PM concentrations, after adjusting for individual and areal characteristics. During eight years of follow-up, 1487 people died with any of 17 cancer types. Lung cancer death was the highest, followed by liver and stomach cancer. Although we did not find the association for all cancer types, possibly because of limited cancer cases, HRs of PM_2.5_ were relatively high for lung, stomach, pancreas, non-Hodgkin’s lymphoma, prostate, esophagus, oral and pharynx, and brain cancer mortality (HRs = 1.44–7.14). High HRs for pancreas, non–Hodgkin’s lymphoma, esophagus, and oral and pharynx cancer were also seen for PM_10_; our findings suggest PM air pollution as a potential risk factor of cancer mortality for upper digestive tracts, mouth, pancreas, and non–Hodgkin’s lymphoma in a highly urbanized population with high exposure to PM for a long time.

## 1. Introduction

Cancer remains one of the major burdens of disease worldwide. In 2016, the World Health Organization reported that about 70% of all deaths are attributed to non-communicable diseases, in which cancer makes up more than 20% [1]. Despite the overall decrease in cancer deaths, owing to early cancer detection and management, the burden of cancer mortality persists, with constant global increase in cancer incidence. For example, the newly diagnosed cancer cases are expected to be 28.4 million cases in 2040, approximately a 50% rise from 2020 [2]. Lung cancer, as the most common cause of cancer death, makes up 18.0% of 9.9 million deaths for the global population, followed by colorectum (9.4%) and liver cancer (8.3%) in 2020 [2]. In order to reduce the heavy burden of cancer, efforts have been devoted to identifying risk factors of cancer. In addition to genetic predisposition, studies reported modifiable behaviors such as smoking, drinking, diet, and physical activity, and recently drew attention to environmental factors [1].

As a prominent environmental risk factor, particulate matter (PM) air pollution has challenged the global community. Many epidemiological and toxicological studies suggested evidence of the causal relationship between long–term exposure to PM and lung cancer incidence and mortality [3]. Owing to cumulative evidence, the International Agency for Research on Cancer (IARC) classified airborne particulate matter as carcinogenic to humans [4]. Most recently, new findings supported the associations with mortality of other cancer types: stomach, colorectal, liver, pancreatic, breast, and bladder cancer [5,6,7,8,9,10,11,12]. However, it is difficult to compare the associations of PM between different cancer types, because most studies examined single cancer types based on different cohorts. Characteristics of cancer and the association with PM may vary depending on the population related to genetic and environmental factors. Furthermore, only a few cohort studies that compared many cancer types were performed, mostly in limited regions of North America and Europe, primarily with low-dose conditions of PM. Findings from low–dose countries may or may not be consistent, when investigated in high-dose conditions.

A population-based cohort in South Korea can provide an opportunity to fill this research gap. The National Health Insurance Service-National Sample Cohort (NHIS–NSC) is a nationwide cohort that makes up of 2.2% of the South Korean population and includes various information from medical utilization and health examination [13]. In South Korea, cancer is the most common cause of death, accounting for 27.5% of the total death [14]. This is greater than the global figure and expected to increase along with population aging and westernized lifestyle [15]. This large portion of cancer deaths can also help elucidate the role of PM air pollution as a risk factor of the various types of cancer. Our previous study used this cohort and found suggestive evidence of the association between PM and lung cancer incidence in the Seoul Metropolitan Area (SMA), where about half of the South Korean population resides [16]. Using the population-based nationwide cohort, this study aims to compare the associations of long-term exposure to PM ≤2.5 or 10 µm in diameter (PM_2.5_ or PM_10_) with cancer mortality by 17 cancer types in the SMA.

## 2. Materials and Methods

### 2.1. Study Population

**Figure 1:**
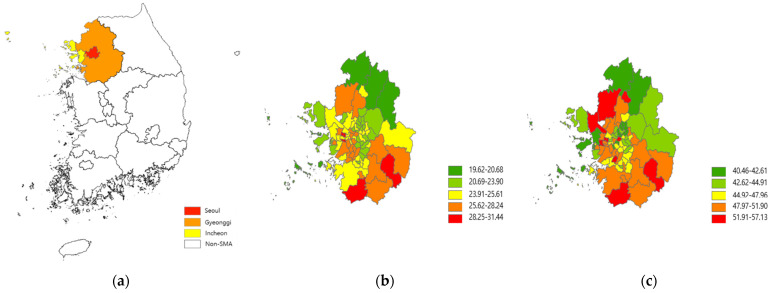
Maps of South Korea and the Seoul Metropolitan Area (**a**), and PM_2.5_ (**b**) and PM_10_ (**c**) concentrations (µg/m^3^) for 2015–2019.

Our study population includes 87,608 subjects selected from the NHIS–NSC. Since South Korea accomplished universal healthcare coverage for all citizens in 2000, the NHIS database has been expanded by containing enormous amounts of information on healthcare utilization and biological and sociodemographic characteristics for the entire population. In 2014, the NHIS created the NHIS–NSC, which includes one million people sampled from the NHIS database and their various individual information for 2002–2015 [13]. We primarily focused on the Seoul Metropolitan Area (SMA), where our previous study found the association between PM and lung cancer incidence [16]. The SMA is a highly urbanized and populated area with relatively high air pollution (48 and 26 µg/m^3^ for annual average concentration of PM_2.5_ and PM_10_, respectively, in 2015), consisting of three regions, namely, Seoul, the capital of South Korea (area = 605 km^2^; population = 10,192,710), Gyeonggi, the most populated province (10,184 km^2^; 11,106,211), and Incheon, the second largest port city (1007 km^2^; 2,664,576) (Figure 1 and Figure S1, see the supplementary file) [17]. A relatively homogenous people, as half of the South Korean population live in the SMA, can help achieve the representativeness of our findings and avoid the difficulty in accounting for the exceptionally heterogeneous subpopulation found in our previous studies [16,18].

From one million people of the NHIS–NSC, we applied our exclusion criteria and selected 87,608 subjects as our study population (Figure S2). To assess the impact of long-term exposure to PM on cancer mortality, we determined the year 2007 as our baseline and employed the exposure period of 5 years. Specifically, we selected study subjects who participated in a health screening covered by the National Health Insurance in 2005–2007, and we followed up their status of cancer death from 2007 to 2015. We restricted our study population to health screenees in order to utilize their various individual characteristics, including biological information, health behavior, and medical history. We additionally excluded those who were younger than the age of 30 or severely disabled, and had no information on cause of death, residential address, or individual characteristics. We did not exclude those who were diagnosed with cancer before the baseline, from 2002 to 2006 (10.44% of the study population), to retain the minimum sample size to investigate each cancer site.

### 2.2. Cancer Mortality and Individual and Areal Characteristics

We identified 17 specific types of cancer mortality based on the International Classification of Disease, 10th reversion (ICD–10): oral and pharynx (C01–C14), esophagus (C15), stomach (C16), colorectal (C18–C21), liver (C22), gallbladder (C23–C24), pancreas (C25), lung (C34), breast (C50), female genitals (C53–C56), prostate (C61), bladder (C67), kidney (C64–66, C68), brain (C71), non-Hodgkin’s (C82–C85), multiple myeloma (C88, C90), and leukemia (C91–C95). We chose these 17 sites, which were investigated in previous studies that compared different cancer sites [6,9] and provided at least 20 cancer deaths in our study population.

For individual-level characteristics, we included age, sex, health insurance premium (0–40%, 40–60%, 60–80%, or 80–100%), employee status, smoking status (never, former or current smoker), smoking period (year), smoking amount (pack per day), frequency of alcohol consumption and physical activity, diet (plant–based, balanced, or meat–based diet), BMI, and family history of any cancer. A health insurance premium is computed based primarily on income and property/asset, regardless of age and health status, and was often used as an indicator for socioeconomic status in previous studies, South Korea [16,18,19]. For area–level characteristics, we used district–specific characteristics for demographics, socioeconomic status, health care access, and area type. Six area-level characteristics represent socioeconomic characteristics and health care accessibility. Demography–related characteristics such as the proportion of the elderly and population density were also often used as indicators of area-level socioeconomic status in South Korea, based on high poverty rate and low economic activity of the elderly population and low population density correlated with large elderly population [20,21,22]. Gross regional domestic product (GRDP) is estimated based on production, distribution, and expenditure of income of each district, indicating local economic activity [23]. We used the proportion of health screening program recipients to represent health care accessibility. Using the district-specific characteristics obtained from the year 2005 Census and general national statistics in 2005, we categorized the elderly population (≥65 years), high school graduates, population size, and gross regional domestic production to the quartiles across districts. We obtained health screening program recipients for each district from the National Health Insurance Statistic in 2008 and computed the rate of recipients. The area types defined as urban, suburban and rural areas were obtained from the Korea Statistics.

### 2.3. Exposure Assessment

We estimated individual–level long–term concentrations of PM using two previously developed prediction models. For PM_10_, we used the national–scale exposure prediction model developed in a universal kriging framework along with more than 300 geographical variables and air pollution regulatory monitoring data [24]. This point–wise spatial model allowed us to predict annual–average concentrations of PM_10_ at any location in South Korea. As address information of NHIS-NSC subjects is available at the district level for maintaining confidentiality, we predicted annual-average PM_10_ concentrations at all census tract centroids and averaged to each district to compute population-representative district averages. Each Metropolitan City or Province includes 5–48 districts with a total of 251–263 districts for 2002–2015 (average and range of area size in 2007 for South Korea: 429 and 3–1818 km^2^; SMA: 149 and 7–878 km^2^). For PM_2.5_, we applied the ratio-based model because nationwide PM_2.5_ regulatory monitoring data are available only after 2015, as opposed to PM_10_, which is available from 2001 [25]. This model provided district-level PM_2.5_ annual-average concentrations based on the ratios of PM_2.5_ to PM_10_ multiplied by district–level PM_10_ predictions. As PM_2.5_ monitoring data are available for 2001–2015 in Seoul, we computed ratios each year for 2001–2015 in Seoul and adjusted regional differences of ratios using the proportions of ratios in Seoul to those in other regions in 2015. Since accumulating evidence has showed stronger associations of various health endpoints with PM_2.5_ than with PM_10_, and since our prediction approach for PM_2.5_ based on ratios showed good model performance [26], we treated the PM_2.5_ analysis as our primary. Finally, we computed the averages of district-level PM_10_ or PM_2.5_ concentrations for each of the previous 5 years over the follow–up period as individual–level long–term exposure to PM. In addition, we computed 1– and 3–year average concentrations for our sensitivity analyses.

### 2.4. Statistical Analysis

We used the time-dependent Cox proportional hazards model to estimate hazard ratios (HRs) and 95% confidence intervals (95% CIs) of cancer mortality per 10 μg/m^3^ increase in PM concentrations, according to each of 17 cancer types. Time–dependent Cox regression allows us to assess the overall risk estimate by combining the survival pattern associated with exposure within each time window of one year for the entire follow-up period as a weighted average of estimates across time windows [27]. The survival time of each participant was calculated from the baseline, 1 January 2007, to the earliest date of the end of the study, 31 December 2015, death, and drop-out. Participants who were lost to follow up, alive at the end of study, or died with other causes were treated censored.

We developed three progressively–adjusted health analysis models to investigate the association between long-term exposure to PM and cancer mortality according to the progressive adjustment of potential confounders. Model 1 included age and sex in addition to PM. In model 2, we added individual-level characteristics. These characteristics were commonly included as confounders in previous cohort studies of long-term air pollution and mortality [28,29]. In model 3, we additionally adjusted for area-level characteristics to account for possible area-level confounding, in addition to individual-level confounding, suggested in our previous studies of PM and mortality in South Korea [16,18,30]. Since the SMA has relatively similar area-level characteristics, we chose model 2 as our primary approach. For our sensitivity analysis, we used 1- and 3-year average concentrations of PM and compared to our primary analysis using average exposure for the previous 5 years. Second, we performed the same analyses using the 206,717 NHIS–NSC subjects for South Korea and compared to our findings for the SMA. Third, we defined cancer deaths based on single-site cancers and compared those to our primary findings, including both single– and multiple–site cancers. Lastly, we excluded those who were diagnosed with cancer before the baseline and compared this to our primary analysis findings.

This study was approved by the Institutional Review Board of the National Cancer Center (IRB code NCC2018–0017).

## 3. Results

### 3.1. Study Population and Individual Characteristics

In 87,608 NHIS–NSC subjects living in the SMA, most participants were middle-aged adults (46.58 years old on average) and more than half of participants had never smoked (67.43%), never or rarely consumed alcohol (54.73%), did not exercise (50.60%), and lived in urban areas (84.46%) (Table 1). People who died with any cancer during the eight years of follow-up for 2007–2015 were likely to be older (58.60 years old), former or current smokers (40.75%) with longer cigarette smoking period for ≥30 years (21.75%), and alcohol consumers drinking almost every day (6.78%) at baseline, compared to those who survived to the end of the study, were lost, or died with other causes (46.36 years old, 32.42%, 5.71%, and 3.05%, respectively). These patterns in the total SMA population and subpopulations by cancer death status were similar for 206,717 people in the entire country (Table S1).

### 3.2. Study Population and Cancer Mortality

For eight years of follow-up in our study, from 2007 to 2015, 1563 people (782,090 person years; 1.78%) died with any cancer in 87,608 NHIS–NSC SMA subjects, while 1487 died with any of 17 cancer types. According to the cancer site, lung cancer gave the highest deaths (23.50%), followed by liver cancer (13.57%) and stomach cancer (12.68%) (Table 2). This pattern was similar in 206,717 subjects over South Korea (24.81, 14.93, and 12.50%, respectively) (Table S2).

Individual characteristics in 2005–2007 showed similarities and differences across 17 cancer types (Table 2). Breast and prostate cancer mortality showed the lowest and highest mean ages (48.95 and 64.39 years old, respectively). Males are more likely to die with all types of cancer than females, except only for brain cancer (41.67%). In particular, more than 90% of esophagus and oral and pharynx cancer deaths occurred in males. Additionally, people in the lowest 25% for health insurance premiums tended to die more with lung, stomach, colorectal, kidney, and bladder cancer (>30%), while people in the highest 25% for health insurance premiums showed higher mortality with liver, pancreas, gallbladder, prostate, and esophagus cancer (>30%). Lastly, a higher percent of ever smokers was found in lung, prostate, esophagus, and oral and pharynx cancer mortality (>50%). Such trends are mostly similar in South Korea with a few exceptions (Table S2). For example, oral and pharynx cancer showed different patterns between low and high health insurance premiums in the SMA (8.00% and 24.00%), but there was greater similarity in the South Korean population (21.21 and 22.73%).

### 3.3. Association between Long-Term PM Exposure and Cancer Mortality

**Figure 2:**
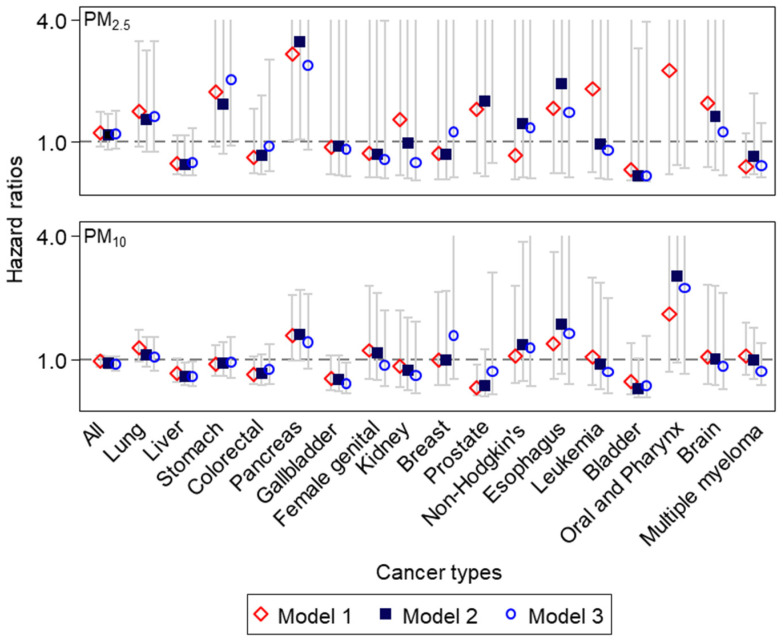
Hazard ratios and 95% confidence intervals of cancer-specific mortality for a 10μg/m^3^ increase in individual-level PM concentrations for the previous 5 years, after adjusting for individual or areal characteristics in 87,608 National Health Insurance Service–National Sample Cohort subjects in the Seoul Metropolitan Area (cancer types seen from the highest number of deaths on the left to the lowest on the right; the maximum y–axis limit set to 4.0, for improving readability without presenting extremely high upper bounds of confidence intervals, as seen in Tables S3 and S4).

Figure 2 shows HRs and 95% CIs of cause-specific cancer mortality for a 10 μg/m^3^ increase in PM_2.5_ or PM_10_ concentrations for the previous five years in the SMA population. Although both PM_2.5_ and PM_10_ showed HRs close to 1 for all cancer mortality (HR = 1.16, 95% CI = 0.79–1.69), HRs somewhat varied across 17 cancer types (Table S3). For PM_2.5_, lung cancer showed a positive estimate (HR = 1.55, 95% CI = 0.73–3.26). HRs were particularly high for stomach, pancreas, non-Hodgkin’s lymphoma, prostate, esophagus, oral and pharynx, and brain cancer mortality (HRs = 1.44–7.14 for non-Hodgkin’s to oral and pharynx cancer mortality). These patterns were mostly similar for PM_10_, with some exceptions of negative estimates for stomach and prostate cancer. Nonetheless, effect estimates of all 17 cancer types were statistically non-significant, mainly due to small numbers of cancer deaths.

Our sensitivity analysis for the entire country showed similarities and differences from those for the SMA (Figure S3 and Tables S5 and S6). Stomach, prostate, non-Hodgkin’s lymphoma, esophagus, and brain cancer that showed higher HRs of PM_2.5_ compared to other cancer types in the SMA also showed positive and higher estimates (HRs = 1.12–1.87) (Table S5). Different from the SMA, in South Korea, leukemia showed the highest estimate (HR = 2.39, 95% CI = 0.84–7.37), whereas lung cancer gave a negative estimate (HR = 0.82, 95% CI = 0.63–1.08). None of these estimates were statistically significant.

Our sensitivity analyses, using PM_2.5_ for the previous 1 or 3 years, generally showed consistent results with those of the main analysis using 5 years of exposure in the SMA. HRs were positive for lung (1.68 and 1.62, 1 year and 3 year HRs, respectively), stomach (1.43 and 1.92), pancreas (2.47 and 3.60), prostate (2.73 and 3.57), esophagus (3.02 and 2.87), and oral and pharynx (8.14 and 5.31) cancer mortality (Table S3). However, gallbladder, female genital, non-Hodgkin’s lymphoma, leukemia, and brain cancer mortality showed the different patterns across shorter and longer exposure periods of averaging. The analysis using single–site definition for cancer mortality also showed consistent results with those of the main analysis, including single– and multiple–site cancers (Table S7). Exclusion of those who were diagnosed with cancer before the baseline also showed generally consistent results with our primary findings (Table S8).

## 4. Discussion

Using a population-based well-established cohort, we focused on 17 different types of cancer and compared the associations of cancer mortality with long–term exposure to PM in a highly urbanized population exposed to a relatively high level of PM air pollution. Although we did not find statistical significance for the association across all cancer types possibly resulting from the limited cancer cases, our findings suggest potentially higher mortality risk of PM_2.5_ in lung, stomach, pancreas, prostate, non–Hodgkin’s lymphoma, esophagus, oral and pharynx, and brain cancer compared to other cancer types. The suggestive high risks of pancreas, Non–Hodgkin’s lymphoma, esophagus, and oral and pharynx cancer were consistent for PM_10_.

This study adds an important understanding of the variation in the association of PM and cancer mortality across different cancer sites under a high-dose environment of PM. Most previous cohort studies of PM have focused on lung cancer [3]. Although the mutagenic, epigenetic, and inflammatory mechanisms are shared largely with all types of cancer, it is only recently that large cohort studies have begun to expand to other cancer types [27,31,32,33,34,35,36,37]. Furthermore, there have only been a few studies that compared the associations across different types of cancer and identified the specific sites more affected by PM air pollution than others. The findings of these studies are still too inconsistent to provide evidence of the difference in the association [3]. To our knowledge, at least three studies paid attention to different cancer types and investigated PM as a risk factor of mortality in a generally healthy population: two studies in the U.S. and one in Hong Kong. The lack of evidence could possibly result from the unavailability of representative and sufficient cancer data for different sites. Our study aimed to fill in this research gap, using a representative population exposed to a high-dose of PM from a population-based cohort, constructed from the national health insurance database, taking advantage of a universal health care system.

Previous studies that compared the associations of PM and cancer mortality across various cancer types showed inconsistent findings, possibly derived by different environmental and/or population characteristics. Two nationwide studies in the U.S. examined 22 cancer sites in 635,539 subjects (18–84 years) of the Public National Health Interview Survey (NHIS) for 1987–2014 and 29 cancer sites in 623,048 adults (≥30 years) of the American Cancer Society Cancer Prevention Study II (ACS–CPS II) cohort for 1982–2004 [6,9]. Both studies found the associations of PM_2.5_ with colorectal cancer, and reported largely positive but non–significant HRs for cancers of the esophagus and female genitals (Table 3). Some differences were also found. The ACS–CPS II study reported the association with kidney cancer which showed a non-significantly negative estimate in the NHIS cohort. The association was found for mouth, stomach, breast, female genital, non–Hodgkin’s lymphoma, and leukemia in the NHIS study but not in the ACS–CPS II study. These differences could be related to different population characteristics and study period. The NHIS population of 635,539 subjects were young and old adults aged 18–84 years, living in the continental U.S. including suburban and rural areas in addition to urban areas, and participated from late 1980s through 2010s. In contrast, the ACS–CPS II population of 623,048 subjects were adults over 30 years, mostly from urban areas, followed up from the 1980s through to the early 2000s. The other comparison study, performed in Hong Kong with a much higher level of PM_2.5_ (33.7 µg/m^3^ compared to 10.7 and 12.6 µg/m^3^ in two U.S. studies), and an older population, aged ≥ 65, also showed differences in their findings. This cohort of 66,820 people showed the associations with cancers of breast and upper digestive tracts including esophagus and stomach, and digestive accessary organs including liver, gall bladder, and pancreas (Table 3). Their high effect estimates for upper digestive tracts are also seen in our study as another Asian population, whereas their findings for breast and female genital cancers are not replicated in our study. Our findings are even more different from the findings of two U.S. studies. Except esophagus cancer, which consistently showed higher effect estimates, all the other cancers of the breast, female genitals, and bladder found associated in the U.S. studies showed no associations with HRs close to 1 in our study. In addition to the differences in pollution sources, physical environments, and population, another possible explanation for this inconsistency could be the much smaller sample size compared to the two U.S. studies.

Our findings of suggestive risk of stomach, pancreas, and oral and pharynx cancer mortality for exposure to long–term PM_2.5_ was also found in some previous cohort studies that investigated mortality or incidence of single cancer types (Figure S4). A study of 400,000 adults in Taiwan reported the association of PM_2.5_ and stomach cancer mortality [33], while a European cohort study of 305,551 adults in 10 countries showed the association with incidence of stomach cancer [34]. A U.S. cohort study based on a multi-ethnic population in the Los Angeles area reported the association with incidence of pancreas cancer [38]. We did not find any studies that reported the association with non-Hodgkin’s lymphoma which showed a high HR in our study. As the incidence of non-Hodgkin’s lymphoma has recently increased in many countries including South Korea [39], our finding provides a motivation to explore new cancer types. Different from our findings, increasing numbers of individual cohort studies examined bladder, kidney, breast, and brain cancers and found the associations. While recent review studies of breast and brain cancers concluded that the evidence still remains inconsistent [40,41], review studies of bladder and kidney cancer concluded relatively consistent evidence [42,43]. Future studies should include extended populations to investigate these cancers with relatively low incidence and mortality.

In our study, all of our estimates were statistically non-significant possibly because of small cancer cases. Although we relied on a population-based cohort, one million cohort is still hampered by limited power when we applied our conservative inclusion criteria and pruned to about 90,000 people as our study population. To address the issue of low power, we performed a sensitivity analysis by extending the study population to the entire country. However, this nationwide analysis also gave mostly non-significant effect estimates. These uncertain and less stable effect estimates may be derived by the heavily diverse population from different regions according to socioeconomic characteristics or environmental factors, which were not sufficiently adjusted by area-level variables included in our analyses. Future studies can include further extended populations fully leveraging the national health insurance database. In addition, investigation of cancer incidence could have improved the lack of power. However, we focused on cancer mortality in our study to avoid potential outcome measurement errors in incidence and the impact of different degree of error across different cancer types on the comparison. Future studies should expand the comparison across cancer sites to cancer incidence. Lastly, we did not account for multiple comparison of 17 cancer types, because our findings gave large uncertainty derived by small cases. Future research using extended populations should apply multiple comparison correction.

Our study includes some limitations to provide topics for future research. Our study used district-level PM concentrations as individual exposure based on limited address availability. In addition, we relied on exposure to outdoor PM and did not take into account indoor and/or personal exposure. These limitations may lead to exposure measurement errors that affect accuracy and/or precision in subsequent health effect analyses [43,44]. Although infiltration of PM is higher compared to gaseous pollutants [45,46], indicating the small impact of measurement errors when outdoor PM is used, emerging new technology such as portable sensors can help assess indoor PM of cancer patients [46]. Future studies should improve exposure assessment by using detailed address information and incorporating indoor and personal exposure. In addition, we obtained all individual–level and area-level variables at baseline to maximize the sample size, but some of these characteristics such as BMI could vary over time. Further studies should apply time-varying information and confirm our findings. Lastly, we did not exclude cancer patients, comprising 10% of our population, in order to attain sufficient sample sizes to investigate the association by 17 different cancer sites. This inclusion would make it difficult to distinguish between the impact of PM on cancer occurrence and its impact on progression, although our sensitivity analysis showed generally consistent findings with our primary results including patients. Differences in severity and induction period depending on the cancer type could make this impact even more complicated. Future studies should clarify this impact in extended populations.

## 5. Conclusions

Although our study did not find associations, findings suggested PM air pollution as a potential risk factor of deaths for pancreas, non–Hodgkin’s lymphoma, esophagus, and oral and pharynx cancer other than lung cancer in an urban population exposed to high levels of PM air pollution. Further studies need to investigate the difference in the association by cancer types using extended populations and refined exposure measurement.

## Figures and Tables

**Table 1:** Descriptive summary of individual and areal characteristics of 87,608 National Health Insurance Service–National Sample Cohort subjects and by their cancer death status in the Seoul Metropolitan Area (SMA) for 2005–2007.

Characteristic	Variable	Value	Total (87,608)	Cancer Death ^1^	
				No (86,045)	Yes (1563)
Demography	Age (year)		46.58 (11.01)	46.36 (10.89)	58.60 (11.18)
	Sex	Male	53.83	53.57	67.95
Socioeconomic status	Health insurance premium	0–40%	24.66	24.56	30.20
		40–60%	22.10	22.17	18.62
		60–80%	24.72	24.78	21.37
		80–100%	28.51	28.49	29.81
	Employed	Yes	38.20	38.47	23.67
Behavior	Cigarette smoking status ^2^	Never	67.43	67.58	59.25
		Former	9.82	9.78	12.28
		Current	22.75	22.64	28.47
	Cigarette smoking amount (packs per day) ^2^	Never	74.99	75.12	67.64
		<0.5	5.24	5.17	8.69
		0.5–1	13.68	14.62	16.65
		1–2	5.74	5.72	6.94
		≥2	0.35	0.36	0.07
	Cigarette smoking period (year) ^2^	Never	69.31	69.44	61.98
		<5	1.63	1.64	1.27
		5–9	2.87	2.90	1.54
		10–19	11.68	11.77	6.69
		20–29	8.51	8.54	6.76
		≥30	5.99	5.71	21.75
	Alcohol consumption	Never or rarely	54.73	54.67	57.77
		2–3 per month	16.88	16.97	12.22
		1–2 per week	18.33	18.41	14.08
		3–4 per week	6.94	6.90	9.15
		Almost everyday	3.12	3.05	6.78
	Physical activity	None	50.60	50.55	53.49
		1–2 per week	27.93	28.05	21.56
		3–4 per week	12.50	12.53	10.68
		5–6 per week	3.08	3.08	2.69
		Almost everyday	5.89	5.79	11.58
	Nutrition	Plant–based diet	21.74	21.69	24.29
		Balanced diet	74.22	74.26	72.1
		Meat–based diet	4.04	4.05	3.61
	BMI ^3^	<18.5	2.43	2.40	4.09
		18.5–25	62.59	62.6	62.19
		25–30	31.73	31.76	30.13
		≥30	3.25	3.25	3.58
Family history	Cancer	Yes	15.15	15.20	12.54
Area-level	Elderly population	0–25%	23.64	23.71	19.83
		25–50%	27.85	27.87	26.94
		50–75%	23.33	23.37	21.37
		75–100%	25.17	25.05	31.86
	High school graduates	0–25%	25.40	25.35	28.47
		25–50%	25.36	25.38	24.25
		50–75%	23.71	23.75	21.63
		75–100%	25.53	25.52	25.66
	Gross regional domestic product	0–25%	24.78	24.77	25.72
		25–50%	16.12	16.13	15.80
		50–75%	41.74	41.70	43.89
		75–100%	17.35	17.40	14.59
	Population density	0–25%	24.24	24.19	27.32
		25–50%	25.19	25.25	21.94
		50–75%	24.16	24.16	24.18
		75–100%	26.40	26.40	26.55
	Area type	Urban	84.46	84.54	80.23
		Suburban	12.21	12.16	14.4
		Rural	3.33	3.29	5.37
	Health screening participation rate		58.99 (3.38)	59.00 (3.38)	58.70 (3.30)

^1^ All numbers are presented as percent except for age and health screening participation rate presented as mean (standard deviation). ^2^ Questionnaire did not include the type of cigarette. ^3^ BMI, body mass index.

**Table 2:** Numbers of deaths by 17 cancer types and descriptive summary of individual characteristics in 87,608 National Health Insurance Service-National Sample Cohort subjects in the Seoul Metropolitan Area for 2007–2015.

Cancer Type ^1^	Case	%	Age (Mean (sd))	Sex (Male %)	Premium (Low ^2^ %)	Premium (High ^3^ %)	Ever Smoker (%)
All cancer	1563	100.00	58.66 (11.19)	67.95	30.20	29.81	40.75
Total	1487	95.14	58.60 (11.18)	68.12	30.53	28.98	41.22
Lung	367	23.50	60.39 (9.90)	78.20	38.15	27.52	56.40
Liver	212	13.57	55.33 (11.07)	77.36	25.00	30.66	45.28
Stomach	198	12.68	58.98 (11.93)	64.65	32.32	27.78	33.84
Colorectal	151	9.67	59.23 (10.63)	64.24	35.10	28.48	39.07
Pancreas	129	8.26	60.29 (9.93)	58.91	28.68	34.11	37.98
Gallbladder	69	4.42	61.55 (9.85)	56.52	23.19	44.93	30.43
Female genital ^4^	50	3.20	54.14 (12.44)	0.00	28.00	28.00	0.00
Kidney	38	2.43	55.61 (11.77)	84.21	31.58	26.32	39.47
Breast ^4^	37	2.37	48.95 (9.67)	0.00	24.32	21.62	2.70
Prostate ^5^	36	2.30	64.39 (1.55)	100.00	25.00	30.56	55.56
Non–Hodgkin’s	35	2.24	62.31 (9.00)	71.43	25.71	28.57	34.29
Esophagus	35	2.24	58.23 (9.93)	91.43	20.00	34.29	60.00
Leukemia	32	2.05	55.84 (14.09)	78.13	25.00	12.50	37.50
Bladder	27	1.73	64.70 (10.41)	81.48	33.33	29.63	44.44
Oral and Pharynx	25	1.60	51.44 (12.43)	96.00	8.00	24.00	52.00
Brain	24	1.54	53.54 (12.35)	41.67	25.00	25.00	12.50
Multiple myeloma	22	1.41	60.41 (12.50)	72.73	27.27	13.64	22.73

^1^ The list of cancer types in descending order according to the number of cancer death. ^2^ Lowest 25% of health insurance premium. ^3^ Highest 25% of health insurance premium. ^4^ Summaries among women. ^5^ Summaries among men.

**Table 3:** Findings of the present study and three previous studies that compared the associations of PM_2.5_ and cancer mortality across different cancer types.

		Shin et al. (2021)		Coleman et al. (2020)		Turner et al. (2017)		Wong et al. (2016)	
Characteristics									
Country		South Korea		USA		USA		Hong Kong	
Population size		87,608		635,539		623,048		66,820	
Study period		2002–2015		1987–2014		1982–2004		1998–2001	
Cancer types ^1^	ICD10	Case ^2^	HR (95% CI)	Case ^2^	HR (95% CI)	Case ^2^	HR (95% CI)	Case ^2^	HR (95% CI)
Oral and pharynx	C01–C14	25	7.14 (0.40–126.86)	374	1.19 (0.74–1.91)	262	1.03 (0.84–1.26) ^3^	—	—
						58	0.93 (0.61–1.44) ^4^	—	—
						243	0.88 (0.70–1.10) ^5^	—	—
Esophagus	C15	35	2.43 (0.21–27.82)	599	0.59 (0.38–0.90)	1180	1.02 (0.93–1.13)	323	1.42 (1.06–1.89) ^6^
Stomach	C16	198	1.90 (0.70–5.17)	525	1.87 (1.20–2.92)	1340	1.00 (0.91–1.09)		
Colorectal	C18–C21	151	0.64 (0.19–2.13)	2572	1.29 (1.05–1.58)	6475	1.04 (1.00–1.08)	719	1.01 (0.79–1.30)
Liver	C22	212	0.42 (0.16–1.15)	761	1.32 (0.94–1.85)	1003	1.05 (0.94–1.16)	676	1.35 (1.06–1.71) ^7^
Gallbladder	C23–C24	69	0.89 (0.16–4.90)	—	—	403	1.03 (0.87–1.22)		
	
Pancreas	C25	129	3.47 (1.05–11.51)	1607	1.09 (0.83–1.44)	3812	0.98 (0.92–1.03)		
Lung	C33–C34	367	1.55 (0.73–3.26)	7420	1.13 (1.00–1.26)	—	—	1408	1.14 (0.96–1.36)
	
Breast	C50	37	0.67 (0.06–7.50)	2099	1.33 (1.08–1.64)	3844	1.03 (0.97–1.08)	111	1.80 (1.26–2.55)
Female genital	C53–C56	50	0.69 (0.10–4.66)	750	1.20 (0.73–1.96)	611	1.04 (0.91–1.19) ^8^	138	1.73 (1.17–2.54) ^11^
				237	1.77 (1.00–3.16)	115	1.34 (0.98–1.83) ^9^		
				392	1.03 (0.69–1.53)	987	1.03 (0.93–1.15) ^10^		
Prostate	C61	36	1.98 (0.13–29.76)	1215	0.91 (0.68–1.22)	1068	0.96 (0.86–1.06)	143	1.02 (0.51–2.04) ^12^
Bladder	C67	27	0.16 (0.01–4.37)	589	1.48 (1.00–2.20)	1324	1.13 (1.03–1.23)	155	0.98 (0.58–1.64) ^14^
Kidney	C64–C66, C68	38	0.94 (0.09–10.07)	603	0.98 (0.66–1.46) ^13^	927	1.14 (1.03–1.27)		
Brain	C71	24	1.62 (0.29–8.97)	622	1.48 (0.96–2.29)	1591	1.04 (0.96–1.14)	—	—
	
Non-Hodgkin’s	C82–C85	35	1.44 (0.12–17.83)	1016	1.48 (1.10–1.98)	2840	1.00 (0.94–1.07)	310	1.29 (0.86–1.95) ^15^
Multiple myeloma	C90	22	0.63 (0.18–2.18)	541	0.99 (0.64–1.53)	1421	0.97 (0.89–1.07)		
Leukemia	C91–C95	32	0.93 (0.07–12.45)	970	1.43 (1.05–1.97)	2584	1.01 (0.94–1.07)		

^1^ The list of cancer types are in descending order according to ICD10. ^2^ Number of cases. ^3^ Tongue and mouth, C01–C06; ^4^ salivary gland, C07–C08; ^5^ pharynx, C09–C14; ^6^ upper digestive tract, C15–C16; ^7^ accessory organs, C22–C25; ^8^ uterus, C54–C55; ^9^ cervix, C53; ^10^ ovary, C56; ^11^ female genital, C51–C58; ^12^ male genital, C60–C63; ^13^ kidney, C64–C65; ^14^ urinary, C64–C68; ^15^ lympho-hematopoietic, C81–C96.

## Supplementary Materials

The following are available online at https://www.mdpi.com/article/10.3390/ijerph19063180/s1, Figure S1: Maps of South Korea and the Seoul Metropolitan Area (a), and district-specific concentrations of PM_2.5_ (b) and PM_10_ (c) for 2015-2019, Figure S2: Flow chart of our application of subject exclusion criteria to the National Health Insurance Service-National Sample Cohort (NHIS-NSC) subjects, Table S1: Descriptive summary of individual and areal characteristics of 206,717 National Health Insurance Service-National Sample Cohort subjects and by their cancer death status in the South Korea for 2005-2007, Table S2: Numbers of deaths for 17 cancer types and descriptive summary of individual characteristics in 206,717 National Health Insurance Service-National Sample Cohort subjects in South Korea for 2007-2015, Figure S3: Hazard ratios and 95% confidence intervals of cause-specific mortality for an increase of 10μg/m^3^ in individual-level PM concentrations for the previous 5 years after adjusting for individual or areal characteristics in 206,717 National Health Insurance Service-National Sample Cohort subjects in South Korea (cancer types seen from the highest number of deaths on the left to the lowest on the right; the maximum y-axis limit set to 3.0, for improving readability without presenting extremely high upper bounds of confidence intervals as seen in Table S5), Table S3: Hazard ratios and 95% confidence intervals of cancer-specific mortality for a 10μg/m^3^ increase in individual-level PM_2.5_ concentrations for the previous 5 years after adjusting for individual or areal characteristics in 87,608 National Health Insurance Service-National Sample Cohort subjects in the Seoul Metropolitan Area, Table S4: Hazard ratios and 95% confidence intervals of cancer-specific mortality for a 10μg/m^3^ increase in individual-level PM_10_ concentrations for the previous 5 years after adjusting for individual or areal characteristics in 87,608 National Health Insurance Service-National Sample Cohort subjects in the Seoul Metropolitan Area, Table S5: Hazard ratios and 95% confidence intervals of cause-specific mortality for an increase of 10μg/m^3^ in individual-level PM_2.5_ concentrations for the previous 5 years after adjusting for individual or areal characteristics in 206,717 National Health Insurance Service-National Sample Cohort subjects in the South Korea, Table S6: Hazard ratios and 95% confidence intervals of cause-specific mortality for an increase of 10μg/m^3^ in individual-level PM_10_ concentrations for the previous 5 years after adjusting for individual or areal characteristics in 206,717 National Health Insurance Service-National Sample Cohort subjects in the South Korea, Figure S4: Hazard ratios and 95% confidence intervals of mortality or incidence of stomach, pancreas, and oral and pharynx cancer for long-term exposure to PM_2.5_, Table S7: Hazard ratios and 95% confidence intervals of lung, pancreas, non-Hodgkin’s, esophagus, and oral and pharynx cancer mortality for a 10μg/m^3^ increase in individual-level PM_2.5_ concentrations for the previous 5 years in the primary model (model 2) by definition of cancer deaths based on both single and multiple cancer sites versus single sites only in 87,608 National Health Insurance Service-National Sample Cohort subjects in the Seoul Metropolitan Area, Table S8: HRs and 95%CI of lung, pancreas, non-Hodgkin’s, esophagus, and oral and pharynx cancer mortality for a 10μg/m^3^ increase in individual-level PM_2.5_ concentrations for the previous 5, 3, and 1 years in primary model (model 2) for the comparison between including and excluding lung cancer patients before 2006 using National Health Insurance Service-National Sample Cohort subjects in the Seoul Metropolitan Area.
