# Supplementary material for: Different Mortality Risks of Long-Term Exposure to Particulate Matter across Different Cancer Sites
Source: Int J Environ Res Public Health. 2022 Mar 8;19(6):3180. doi: 10.3390/ijerph19063180 (PMC8951617; doi:10.3390/ijerph19063180)
Supplement: Supplementary file 1 [file ijerph-19-03180-s001.zip › ijerph-1548708-supplementary.pdf]

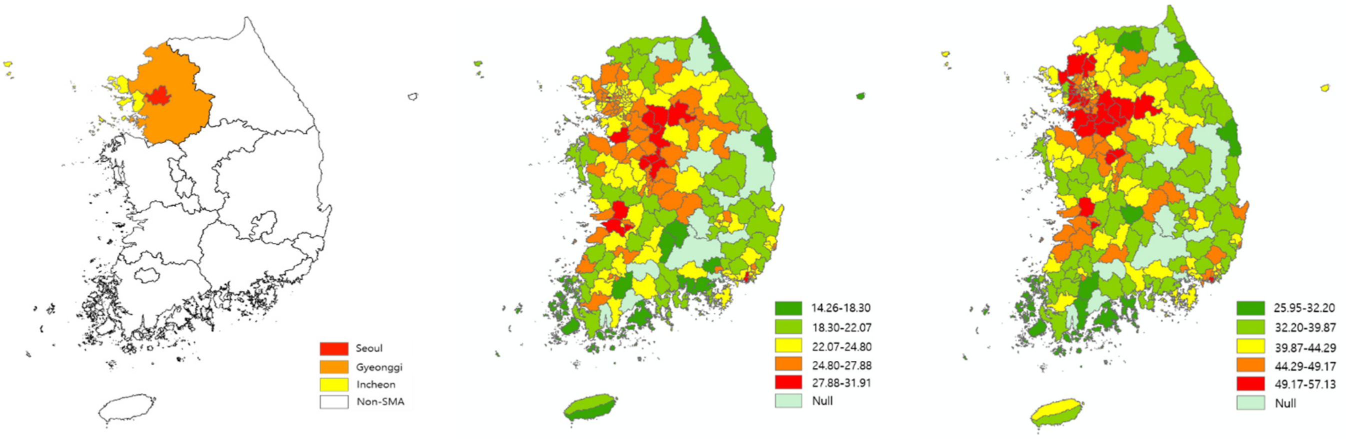

(**a**) (**b**) (**c**)

Figure S1. Maps of South Korea and the Seoul Metropolitan Area (a), and district-specific concentrations of PM2.5 (b) and PM10 (c) for 2015-2019.

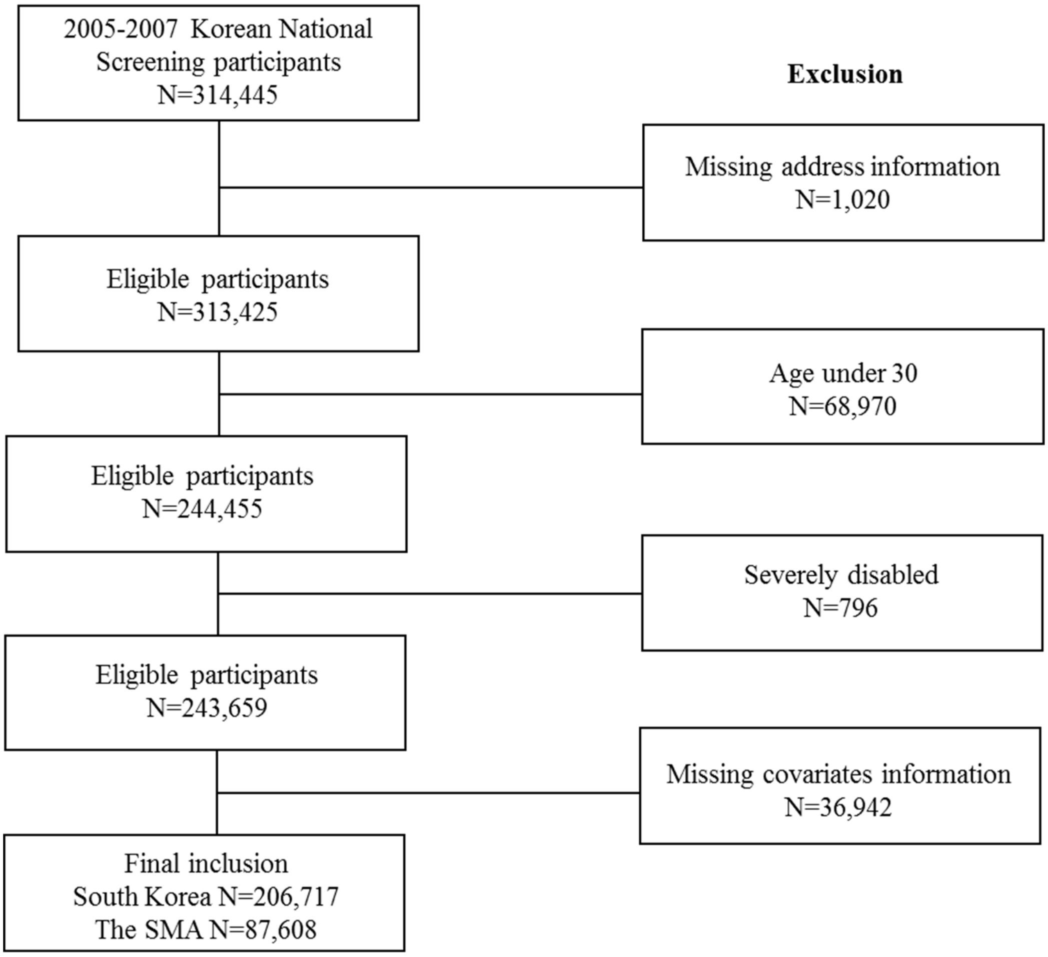

Figure S2. Flow chart of our application of subject exclusion criteria to the National Health Insurance Service-National Sample Cohort (NHIS-NSC) subjects.

Table S1. Descriptive summary of individual and areal characteristics of 206,717 National Health Insurance Service-National Sample Cohort subjects and by their cancer death status in the South Korea for 2005-2007.

Characteristic	Variable	Value	Total (206,717)	Cancer death[1]	
				No (202,356)	Yes (4,361)
Demography	Age		47.76 (11.46)	47.49 (11.33)	60.19 (10.70)
	Sex	Male	52.76	52.41	68.68
Socioeconomic status	Health insurance premium	0-40%	25.58	25.50	29.40
		40-60%	22.18	22.20	20.89
		60-80%	25.10	25.16	22.54
		80-100%	27.14	27.14	27.17
	Employed	Yes	35.29	36.65	18.55
Behavior	Cigarette smoking status[2]	Never smoker	68.82	69.03	59.07
		Former smoker	9.22	9.17	11.69
		Current smoker	21.96	21.90	29.24
	Cigarette smoking amount (pack per day)[2]	Never smoker	76.00	76.19	67.00
		<0.5	5.20	5.10	10.28
		0.5-1	12.97	12.91	16.19
		1-2	5.44	5.43	6.09
		≥2	0.38	0.38	0.44
	Cigarette smoking period (year)[2]	Never smoker	70.70	70.89	61.50
		<5	1.54	1.55	1.15
		5-9	2.57	2.60	1.24
		10-19	10.22	10.45	4.63
		20-29	8.01	8.04	6.69
		≥30	6.85	6.47	24.79

	Alcohol consumption	Never or rarely	57.54	57.47	60.74
		2-3 per month	15.54	15.67	9.52
		1-2 per week	16.90	16.99	12.80
		3-4 per week	6.53	6.49	8.28
		Almost everyday	3.49	3.37	8.67
	Physical activity	None	53.54	53.36	62.05
		1-2 per week	25.87	26.04	17.82
		3-4 per week	11.20	11.27	7.68
		5-6 per week	2.83	2.84	2.38
		Almost everyday	6.56	6.49	10.07
	Nutrition	Plant-based diet	22.05	22.02	23.09
		Balanced diet	74.38	74.38	74.45
		Meat-based diet	3.57	3.59	2.46
	BMI[3]	<18.5	2.46	2.41	5.09
		18.5-25	62.94	62.9	64.6
		25-30	31.52	31.6	27.65
		≥30	3.08	3.09	2.66
Family history	Cancer	Yes	13.72	13.79	10.69
Area-level	Elderly population	0-25%	22.59	25.46	18.64
		25-50%	22.89	26.73	21.67
		50-75%	26.48	23.32	23.60
		75-100%	25.03	24.5	36.09
	High school graduates	0-25%	25.1	24.89	36.12
		25-50%	27.08	26.01	23.55
		50-75%	22.07	24.46	20.34

	75-100%	22.75	24.64	20.00
Gross Regional Domestic Product	0-25%	25.5	24.24	34.01
	25-50%	24.71	25.42	23.82
	50-75%	20.15	19.52	16.79
	75-100%	29.64	30.81	25.38
Population density	0-25%	25.63	24.75	35.77
	25-50%	24.85	24.46	22.68
	50-75%	24.42	24.89	20.66
	75-100%	25.06	25.89	20.89
Area type	Urban	60.03	60.23	50.40
	Suburban	24.67	24.64	26.07
	Rural	15.30	15.12	23.53
Health screening participation rate		61.60 (4.27)	61.61 (4.27)	61.37 (4.22)

[1]All numbers are presented as percent except for age and health screening participation rate presented as mean(standard deviation).

[2]Questionnaire did not include the type of cigarette

[3]BMI, Body Mass Index

Table S2. Numbers of deaths for 17 cancer types and descriptive summary of individual characteristics in 206,717 National Health Insurance Service-National Sample Cohort subjects in South Korea for 2007-2015.

Cancer types[1]	ICD10	Case	%	Age (mean(sd))	Sex (Male %)	Premium (Low[2] %)	Premium (High[3] %)	Ever smoker (%)
All cancer		4,361	100.00	60.26 (10.69)	68.68	29.40	27.17	40.93
Total		4,122	94.52	60.19 (10.70)	69.07	29.28	26.86	41.36
Lung	C34	1,082	24.81	61.97(9.29)	78.56	32.90	25.14	56.10
Liver	C22	651	14.93	56.92 (10.73)	77.42	26.27	25.65	44.09
Stomach	C16	545	12.50	60.68 (11.67)	69.54	30.83	27.71	36.15
Colorectal	C18–C21	404	9.26	61.10 (1045)	61.63	31.44	26.98	36.39
Pancreas	C25	349	8.00	60.88 (9.92)	60.74	27.22	31.52	33.81
Gallbladder	C23–C24	228	5.23	61.66 (9.48)	58.33	21.93	34.65	28.95
Female genital[4]	C53-C56	110	2.52	55.90 (12.43)	0.00	32.73	28.18	2.73
Kidney	C64–C66, C68	84	1.93	60.60 (10.54)	76.19	34.52	23.81	36.90
Breast[4]	C50	75	1.72	48.56 (10.56)	0.00	25.33	21.33	4.00
Prostate[5]	C61	97	2.22	65.99 (8.52)	100.00	26.80	28.87	45.36
Non-Hodgkin's	C82–C85	103	2.36	61.25 (1.63)	64.08	27.18	20.39	33.01
Esophagus	C15	98	2.25	60.61 (8.90)	94.90	30.61	25.51	66.33
Leukemia	C91–C95	73	1.67	55.86 (12.99)	63.01	21.92	20.55	31.51
Bladder	C67	56	1.28	65.16 (9.77)	76.79	26.79	37.50	39.29
Oral and Pharynx	C01–C14	66	1.51	57.82 (12.13)	84.85	21.21	22.73	56.06
Brain	C71	50	1.15	56.22 (11.68)	44.00	26.00	26.00	20.00
Multiple myeloma	C88, C90	51	1.17	60.27 (10.47)	64.71	27.45	27.45	21.57

[1]The list of cancer types in descending order according to the number of cancer deaths

[2]Lowest 25% of health insurance premium
[3]Highest 25% of health insurance premium
[4]Summaries among women
[5]Summaries among men

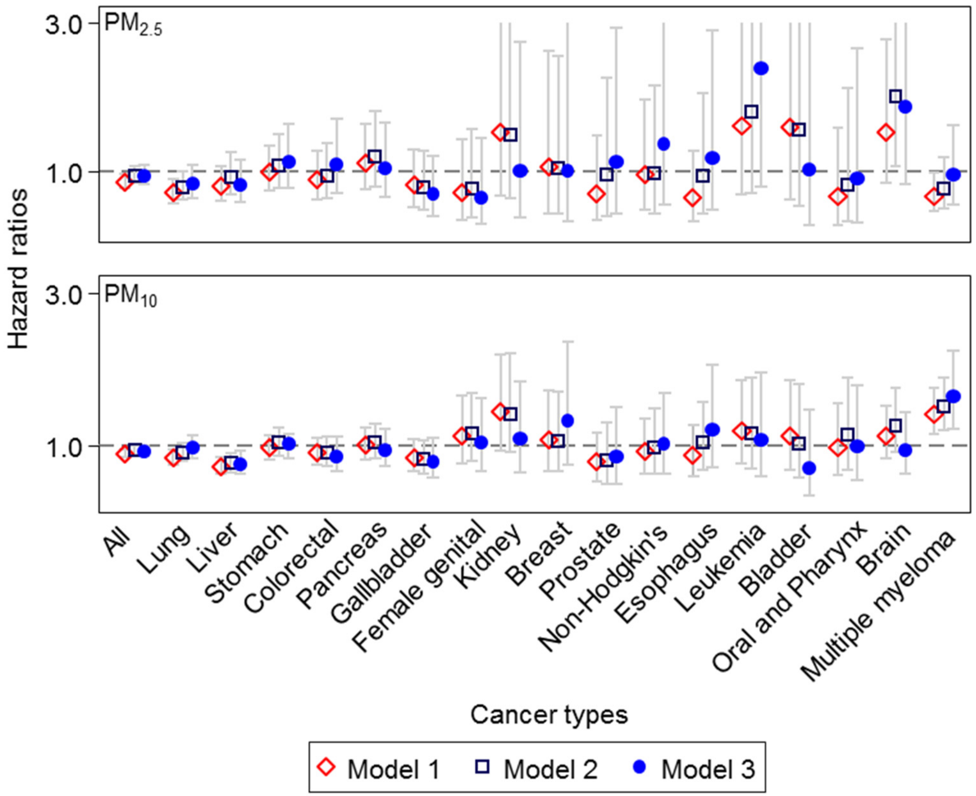

Figure S3. Hazard ratios and 95% confidence intervals of cause-specific mortality for an increase of 10μg/m^3 in individual-level PM concentrations for the previous 5 years after adjusting for individual or areal characteristics in 206,717 National Health Insurance Service-National Sample Cohort subjects in South Korea (cancer types seen from the highest number of deaths on the left to the lowest on the right; the maximum y-axis limit set to 3.0, for improving readability without presenting extremely high upper bounds of confidence intervals as seen in Table S5).

Table S3. Hazard ratios and 95% confidence intervals of cancer-specific mortality for a 10μg/m³ increase in individual-level $PM_{2.5}$ concentrations for the previous 5 years after adjusting for individual or areal characteristics in 87,608 National Health Insurance Service-National Sample Cohort subjects in the Seoul Metropolitan Area.

		Model 1[1]			Model 2[2]*			Model 3[3]		
Cancer type	Exposure period	HRs	LL	UL	HRs	LL	UL	HRs	LL	UL
All cancer	5 year	1.21	0.86	1.72	1.16	0.79	1.69	1.19	0.80	1.75
	3year	1.29	0.92	1.80	1.28	0.90	1.84	1.32	0.91	1.93
	1 year	1.32	0.95	1.83	1.23	0.87	1.75	1.26	0.87	1.81
Lung	5 year	1.74	0.88	3.47	1.55	0.73	3.26	1.61	0.75	3.46
	3year	1.84	0.95	3.58	1.62	0.79	3.31	1.59	0.76	3.36
	1 year	**2.28**	**1.21**	**4.30**	1.68	0.84	3.35	1.65	0.81	3.37
Liver	5 year	0.44	0.17	1.14	0.42	0.16	1.15	0.47	0.17	1.33
	3year	0.47	0.19	1.17	0.45	0.17	1.18	0.50	0.18	1.40
	1 year	0.62	0.25	1.50	0.62	0.24	1.58	0.69	0.26	1.85
Stomach	5 year	2.22	0.88	5.61	1.90	0.70	5.17	2.53	0.90	7.11
	3year	2.07	0.84	5.10	1.92	0.73	5.06	2.46	0.90	6.75
	1 year	1.59	0.66	3.82	1.43	0.56	3.64	1.68	0.64	4.42
Colorectal	5 year	0.60	0.20	1.80	0.64	0.19	2.13	0.88	0.26	3.02
	3year	0.75	0.26	2.17	0.89	0.28	2.83	1.35	0.41	4.47
	1 year	0.74	0.26	2.09	0.89	0.29	2.77	1.33	0.41	4.26
Pancreas	5 year	3.16	1.03	9.75	3.47	1.05	11.51	2.88	0.80	10.30
	3year	3.34	1.14	9.77	3.60	1.16	11.23	3.07	0.89	10.63
	1 year	2.36	0.82	6.82	2.47	0.81	7.59	1.98	0.58	6.72
Gallbladder	5 year	0.86	0.18	4.14	0.89	0.16	4.90	0.81	0.14	4.76
	3year	1.10	0.24	5.02	1.23	0.24	6.32	1.07	0.19	6.00
	1 year	0.92	0.21	4.00	0.80	0.16	3.96	0.68	0.13	3.54
Female genital	5 year	0.69	0.10	4.83	0.69	0.10	4.66	0.56	0.08	3.99

	3year	1.14	0.18	7.12	1.09	0.18	6.66	0.85	0.13	5.68
	1 year	1.21	0.21	7.07	1.16	0.20	6.69	0.97	0.16	6.01
Kidney	5 year	1.53	0.17	14.21	0.94	0.09	10.07	0.47	0.04	5.88
	3year	1.22	0.15	10.22	0.91	0.09	8.83	0.50	0.04	5.80
	1 year	0.74	0.09	5.87	0.61	0.07	5.63	0.36	0.03	3.84
Breast	5 year	0.71	0.06	7.83	0.67	0.06	7.50	1.23	0.11	14.22
	3year	0.88	0.09	8.32	0.84	0.09	8.05	1.54	0.16	14.63
	1 year	0.79	0.10	6.49	0.77	0.09	6.36	1.28	0.16	10.52
Prostate	5 year	1.80	0.21	15.76	1.98	0.13	29.76	6.95	0.45	106.89
	3year	2.70	0.36	20.49	3.57	0.27	46.84	11.79	0.95	145.49
	1 year	2.16	0.28	16.47	2.73	0.20	36.33	7.62	0.64	91.35
Non-Hodgkin's	5 year	0.66	0.07	6.22	1.44	0.12	17.83	1.33	0.09	19.70
	3year	0.46	0.05	4.08	0.79	0.07	9.17	0.69	0.05	9.98
	1 year	0.61	0.07	4.95	0.57	0.06	5.76	0.49	0.04	6.07
Esophagus	5 year	1.81	0.20	16.65	2.43	0.21	27.82	1.70	0.10	29.60
	3year	2.58	0.31	21.73	2.87	0.27	30.72	1.71	0.10	28.08
	1 year	2.47	0.33	18.52	3.02	0.34	27.29	1.74	0.13	24.15
Leukemia	5 year	2.29	0.24	22.01	0.93	0.07	12.45	0.77	0.05	10.99
	3year	1.14	0.12	11.02	0.87	0.07	10.95	0.67	0.05	9.18
	1 year	1.87	0.20	17.41	2.42	0.22	26.90	2.14	0.18	25.88
Bladder	5 year	0.30	0.02	4.07	0.16	0.01	3.29	0.14	0.01	3.94
	3year	0.28	0.02	3.41	0.25	0.01	4.37	0.26	0.01	6.61
	1 year	0.27	0.02	3.17	0.30	0.02	4.54	0.33	0.02	7.27
Oral and Pharynx	5 year	2.76	0.18	41.84	7.14	0.40	126.86	6.74	0.35	131.46
	3year	2.13	0.16	28.19	5.31	0.36	78.93	4.40	0.27	72.45

	1 year	4.34	0.45	42.36	8.14	0.80	83.10	6.84	0.59	78.88
Brain	5 year	1.94	0.36	10.41	1.62	0.29	8.97	1.24	0.16	9.46
	3year	0.72	0.13	4.07	0.72	0.12	4.21	0.70	0.10	4.88
	1 year	1.40	0.24	8.08	1.35	0.23	8.01	1.91	0.28	13.22
Multiple myeloma	5 year	0.37	0.11	1.19	0.63	0.18	2.18	0.39	0.11	1.45
	3year	0.41	0.13	1.32	0.64	0.18	2.24	0.38	0.10	1.36
	1 year	0.41	0.13	1.28	0.59	0.18	1.97	0.38	0.11	1.31

*Primary model

[1]Model 1: PM + age + sex

[2]Model 2: Model 1 + Health insurance premium + Employment status+ Cigarette smoking status + Cigarette smoking amount (pack per day) + Cigarette smoking period (year) + Alcohol consumption + Physical activity + Nutrition + BMI + Family history of cancer

[3]Model 3: Model 2 + district-level of Elderly population, completeness of high school graduates, Gross Regional Domestic Product, and Population density + Area type + Health screening participation

Formula of primary model:

$$\lambda(t) = \lambda_0(t_0)\exp\{\beta_1 PM(t_i) + \beta_2 age(t_0) + \beta_3 sex(t_0) + \beta_4 Health\ insurance\ premium(t_0) + \beta_5 Employment\ status(t_0) + \beta_6 Cigarette\ smoking\ status(t_0) + \beta_6 Cigarette\ smoking\ amount\ (pack\ per\ day)(t_0) + \beta_7 Cigarette\ smoking\ period\ (year)(t_0) + \beta_8 Alcohol\ consumption(t_0) + \beta_9 Physical\ activity(t_0) + \beta_{10} Nutrition(t_0) + \beta_{11} BMI(t_0) + \beta_{12} Family\ history\ of\ cancer(t_0)$$

t_0 *and* t_i: baseline and time *i* between 2007-2015

Table S4. Hazard ratios and 95% confidence intervals of cancer-specific mortality for a 10μg/m³ increase in individual-level PM_{10} concentrations for the previous 5 years after adjusting for individual or areal characteristics in 87,608 National Health Insurance Service-National Sample Cohort subjects in the Seoul Metropolitan Area.

		Model 1[1]			Model 2[2]*			Model 3[3]		
Cancer type	Exposure period	HRs	LL	UL	HRs	LL	UL	HRs	LL	UL
All cancer	5 year	0.96	0.82	1.11	0.93	0.79	1.09	0.88	0.73	1.06
	3year	1.02	0.89	1.18	1.01	0.86	1.17	0.98	0.82	1.18
	1 year	1.06	0.93	1.22	1.03	0.89	1.19	1.02	0.86	1.21
Lung	5 year	1.29	0.96	1.73	1.13	0.82	1.55	1.06	0.74	1.53
	3year	1.29	0.98	1.70	1.14	0.85	1.54	1.07	0.76	1.52
	1 year	**1.36**	**1.05**	**1.77**	1.17	0.88	1.55	1.11	0.80	1.54
Liver	5 year	0.68	0.45	1.02	0.61	0.39	0.94	0.58	0.36	0.95
	3year	0.77	0.52	1.13	0.70	0.46	1.05	0.69	0.43	1.10
	1 year	**0.45**	**0.31**	**0.65**	0.86	0.58	1.27	0.89	0.57	1.39
Stomach	5 year	0.89	0.58	1.34	0.91	0.59	1.42	0.93	0.56	1.55
	3year	0.92	0.62	1.35	0.96	0.63	1.45	0.97	0.60	1.58
	1 year	0.88	0.61	1.28	0.90	0.61	1.35	0.89	0.57	1.40
Colorectal	5 year	0.65	0.41	1.06	0.66	0.39	1.13	0.76	0.42	1.38
	3year	0.77	0.49	1.21	0.82	0.50	1.34	1.00	0.57	1.76
	1 year	0.80	0.52	1.22	0.86	0.54	1.39	1.05	0.62	1.79
Pancreas	5 year	1.59	0.98	2.56	1.62	0.97	2.69	1.42	0.77	2.60
	3year	**1.61**	**1.03**	**2.52**	**1.63**	**1.01**	**2.63**	1.49	0.83	2.66
	1 year	1.52	0.98	2.35	1.53	0.96	2.44	1.37	0.79	2.39
Gallbladder	5 year	0.54	0.26	1.11	0.51	0.24	1.09	0.42	0.19	0.93
	3year	0.68	0.35	1.32	0.66	0.33	1.33	0.54	0.25	1.17
	1 year	0.69	0.37	1.29	0.63	0.32	1.23	0.53	0.25	1.08
Female genital	5 year	1.22	0.54	2.77	1.16	0.51	2.60	0.88	0.35	2.19

	3year	1.36	0.64	2.88	1.29	0.61	2.72	1.03	0.43	2.46
	1 year	1.32	0.63	2.75	1.26	0.61	2.61	1.04	0.46	2.39
Kidney	5 year	0.85	0.33	2.20	0.74	0.27	2.02	0.61	0.19	1.92
	3year	0.86	0.35	2.11	0.79	0.31	2.05	0.68	0.23	2.05
	1 year	0.90	0.38	2.12	0.81	0.33	2.02	0.74	0.26	2.09
Breast	5 year	0.99	0.38	2.63	1.00	0.37	2.66	1.58	0.54	4.62
	3year	1.11	0.45	2.77	1.11	0.44	2.79	1.69	0.64	4.49
	1 year	1.07	0.45	2.57	1.08	0.45	2.59	1.52	0.61	3.84
Prostate	5 year	**0.33**	**0.12**	**0.88**	0.37	0.11	1.25	0.72	0.16	3.11
	3year	0.46	0.18	1.17	0.54	0.17	1.73	1.14	0.29	4.53
	1 year	0.44	0.17	1.10	0.56	0.18	1.75	1.08	0.29	4.08
Non-Hodgkin’s	5 year	1.09	0.42	2.79	1.35	0.48	3.85	1.30	0.36	4.67
	3year	1.09	0.45	2.63	1.26	0.47	3.36	1.23	0.36	4.19
	1 year	1.13	0.49	2.60	1.07	0.43	2.69	0.99	0.32	3.06
Esophagus	5 year	1.38	0.53	3.60	1.86	0.66	5.22	1.62	0.41	6.44
	3year	1.58	0.65	3.81	1.92	0.73	4.99	1.71	0.46	6.26
	1 year	1.59	0.69	3.62	1.95	0.80	4.74	1.77	0.54	5.80
Leukemia	5 year	1.07	0.38	2.99	0.90	0.28	2.87	0.69	0.19	2.50
	3year	0.84	0.31	2.24	0.91	0.31	2.69	0.69	0.20	2.34
	1 year	0.89	0.35	2.28	1.12	0.40	3.12	0.95	0.30	2.98
Bladder	5 year	0.46	0.15	1.40	0.30	0.09	1.04	0.38	0.09	1.57
	3year	0.52	0.18	1.47	0.42	0.13	1.36	0.56	0.14	2.32
	1 year	0.52	0.19	1.46	0.44	0.14	1.39	0.59	0.15	2.34
Oral and Pharynx	5 year	2.10	0.70	6.32	3.02	0.93	9.84	2.73	0.67	11.18
	3year	1.89	0.67	5.33	2.50	0.83	7.54	2.15	0.59	7.87

	1 year	1.09	0.43	2.78	**2.88**	**1.08**	**7.67**	2.68	0.85	8.40
Brain	5 year	1.06	0.40	2.82	1.02	0.37	2.79	0.84	0.27	2.61
	3year	0.78	0.34	1.76	0.82	0.36	1.91	0.83	0.31	2.23
	1 year	1.06	0.52	2.15	1.09	0.53	2.26	1.32	0.56	3.10
Multiple myeloma	5 year	1.09	0.63	1.90	0.98	0.54	1.77	0.73	0.38	1.40
	3year	1.17	0.71	1.92	1.05	0.61	1.79	0.80	0.43	1.47
	1 year	1.15	0.73	1.83	1.04	0.64	1.70	0.85	0.49	1.48

*Primary model

[1]Model 1: PM + age + sex

[2]Model 2: Model 1 + Health insurance premium + Employment status+ Cigarette smoking status + Cigarette smoking amount (pack per day) + Cigarette smoking period (year) + Alcohol consumption + Physical activity + Nutrition + BMI + Family history of cancer

[3]Model 3: Model 2 + district-level of Elderly population, completeness of high school graduates, Gross Regional Domestic Product, and Population density + Area type + Health screening participation

Table S5. Hazard ratios and 95% confidence intervals of cause-specific mortality for an increase of 10μg/m^3 in individual-level $PM_{2.5}$ concentrations for the previous 5 years after adjusting for individual or areal characteristics in 206,717 National Health Insurance Service-National Sample Cohort subjects in the South Korea.

		Model 1[1]			Model 2[2]			Model 3[3]*		
Cancer type	Exposure period	HRs	LL	UL	HRs	LL	UL	HRs	LL	UL
All cancer	5 year	**0.86**	**0.76**	**0.96**	0.94	0.83	1.06	0.94	0.82	1.08
	3year	**0.87**	**0.78**	**0.98**	0.96	0.85	1.09	0.97	0.84	1.11
	1 year	**0.88**	**0.78**	**0.99**	0.94	0.83	1.07	0.95	0.83	1.09
Lung	5 year	**0.71**	**0.57**	**0.88**	**0.78**	**0.62**	**0.99**	0.82	0.63	1.08
	3year	**0.71**	**0.56**	**0.88**	**0.77**	**0.60**	**0.98**	0.80	0.61	1.05
	1 year	**0.76**	**0.60**	**0.95**	0.79	0.61	1.01	0.82	0.62	1.08
Liver	5 year	0.80	0.60	1.06	0.92	0.68	1.25	0.82	0.58	1.16
	3year	0.81	0.61	1.09	0.93	0.68	1.27	0.83	0.59	1.18
	1 year	0.83	0.61	1.12	0.92	0.66	1.26	0.84	0.59	1.19
Stomach	5 year	0.99	0.74	1.34	1.08	0.78	1.50	1.12	0.77	1.63
	3year	1.04	0.76	1.42	1.13	0.81	1.59	1.19	0.82	1.74
	1 year	1.02	0.74	1.40	1.09	0.77	1.54	1.15	0.79	1.68
Colorectal	5 year	0.89	0.62	1.27	0.93	0.63	1.38	1.09	0.70	1.70
	3year	0.91	0.63	1.32	0.96	0.64	1.43	1.14	0.73	1.78
	1 year	0.83	0.57	1.21	0.86	0.57	1.30	1.02	0.65	1.60
Pancreas	5 year	1.12	0.76	1.64	1.20	0.79	1.81	1.04	0.65	1.66
	3year	1.13	0.76	1.68	1.21	0.79	1.84	1.06	0.67	1.70
	1 year	1.06	0.71	1.59	1.12	0.72	1.72	0.98	0.61	1.58
Gallbladder	5 year	0.82	0.51	1.30	0.78	0.48	1.28	0.69	0.40	1.21
	3year	0.89	0.55	1.43	0.84	0.51	1.40	0.75	0.43	1.30
	1 year	0.97	0.59	1.59	0.88	0.52	1.50	0.79	0.45	1.40
Female genital	5 year	0.70	0.35	1.43	0.77	0.38	1.56	0.64	0.29	1.45

	3year	0.73	0.35	1.51	0.79	0.38	1.64	0.69	0.30	1.56
	1 year	0.69	0.33	1.48	0.75	0.35	1.60	0.68	0.30	1.56
Kidney	5 year	1.53	0.68	3.45	1.49	0.63	3.48	1.01	0.37	2.74
	3year	1.72	0.75	3.91	1.62	0.69	3.84	1.20	0.44	3.26
	1 year	1.49	0.65	3.44	1.38	0.57	3.32	1.01	0.36	2.77
Breast	5 year	1.06	0.43	2.61	1.04	0.42	2.56	1.01	0.33	3.15
	3year	0.98	0.38	2.48	0.94	0.37	2.38	0.99	0.31	3.12
	1 year	0.77	0.30	1.97	0.73	0.28	1.87	0.75	0.24	2.35
Prostate	5 year	0.70	0.33	1.47	0.95	0.40	2.25	1.12	0.43	2.93
	3year	0.83	0.39	1.77	1.16	0.48	2.81	1.40	0.54	3.65
	1 year	0.91	0.42	1.99	1.37	0.55	3.40	1.64	0.63	4.31
Non-Hodgkin's	5 year	0.96	0.47	1.96	0.96	0.43	2.15	1.38	0.55	3.43
	3year	0.86	0.41	1.79	0.91	0.40	2.06	1.26	0.50	3.17
	1 year	0.72	0.34	1.53	0.68	0.29	1.59	0.86	0.33	2.22
Esophagus	5 year	0.63	0.31	1.28	0.94	0.43	2.05	1.18	0.48	2.90
	3year	0.72	0.35	1.50	1.09	0.49	2.44	1.38	0.56	3.39
	1 year	0.88	0.42	1.87	1.21	0.53	2.77	1.52	0.61	3.77
Leukemia	5 year	1.61	0.68	3.77	1.80	0.70	4.61	2.39	0.80	7.18
	3year	1.43	0.61	3.36	1.81	0.71	4.63	2.48	0.84	7.37
	1 year	1.55	0.64	3.75	2.09	0.80	5.41	2.94	0.99	8.71
Bladder	5 year	1.59	0.61	4.14	1.56	0.53	4.60	1.02	0.27	3.89
	3year	1.53	0.58	4.01	1.62	0.54	4.84	1.30	0.35	4.89
	1 year	1.24	0.46	3.32	1.26	0.41	3.83	1.01	0.27	3.77
Oral and Pharynx	5 year	0.66	0.27	1.58	0.82	0.31	2.12	0.89	0.30	2.66
	3year	0.62	0.25	1.55	0.79	0.29	2.13	0.92	0.30	2.76

	1 year	0.79	0.31	2.03	1.03	0.37	2.84	1.35	0.45	4.01
Brain	5 year	1.53	0.84	2.78	**2.00**	**1.06**	**3.77**	1.87	0.82	4.26
	3year	1.19	0.67	2.14	1.54	0.83	2.85	1.33	0.61	2.87
	1 year	1.21	0.64	2.28	1.58	0.81	3.09	1.34	0.60	3.00
Multiple myeloma	5 year	**0.66**	**0.45**	**0.98**	0.77	0.50	1.18	0.95	0.55	1.62
	3year	**0.65**	**0.43**	**0.97**	0.75	0.48	1.17	0.96	0.56	1.66
	1 year	**0.65**	**0.42**	**0.99**	0.78	0.49	1.24	1.03	0.59	1.78

*Primary model

[1]Model 1: PM + age + sex

[2]Model 2: Model 1 + Health insurance premium + Employment status+ Cigarette smoking status + Cigarette smoking amount (pack per day) + Cigarette smoking period (year) + Alcohol consumption + Physical activity + Nutrition + BMI + Family history of cancer

[3]Model 3: Model 2 + district-level of Elderly population, completeness of high school graduates, Gross Regional Domestic Product, and Population density + Area type + Health screening participation

Table S6. Hazard ratios and 95% confidence intervals of cause-specific mortality for an increase of 10μg/m^3 in individual-level PM_{10} concentrations for the previous 5 years after adjusting for individual or areal characteristics in 206,717 National Health Insurance Service-National Sample Cohort subjects in the South Korea.

		Model 1[1]			Model 2[2]			Model 3[3*]		
Cancer type	Exposure period	HRs	LL	UL	HRs	LL	UL	HRs	LL	UL
All cancer	5 year	**0.90**	**0.85**	**0.96**	0.94	0.88	1.01	0.93	0.86	1.01
	3year	**0.92**	**0.86**	**0.98**	0.97	0.90	1.03	0.97	0.89	1.05
	1 year	**0.93**	**0.88**	**0.99**	0.97	0.90	1.04	0.97	0.90	1.05
Lung	5 year	**0.85**	**0.75**	**0.95**	0.91	0.80	1.04	0.98	0.84	1.14
	3year	**0.86**	**0.76**	**0.97**	0.92	0.81	1.05	0.98	0.84	1.15
	1 year	0.89	0.78	1.01	0.94	0.82	1.07	1.00	0.86	1.16
Liver	5 year	**0.73**	**0.63**	**0.85**	**0.77**	**0.65**	**0.90**	**0.76**	**0.63**	**0.93**
	3year	**0.73**	**0.62**	**0.85**	**0.76**	**0.64**	**0.91**	**0.78**	**0.63**	**0.95**
	1 year	**0.75**	**0.63**	**0.89**	**0.77**	**0.65**	**0.92**	**0.81**	**0.66**	**0.98**
Stomach	5 year	0.97	0.82	1.13	1.04	0.87	1.23	1.02	0.83	1.16
	3year	0.98	0.83	1.16	1.06	0.89	1.28	1.06	0.89	1.31
	1 year	0.97	0.82	1.15	1.04	0.87	1.25	1.04	0.87	1.28
Colorectal	5 year	0.91	0.75	1.10	0.90	0.73	1.11	0.87	0.67	1.11
	3year	0.93	0.76	1.14	0.94	0.76	1.17	0.93	0.72	1.19
	1 year	0.89	0.73	1.10	0.90	0.72	1.12	0.90	0.70	1.15
Pancreas	5 year	1.00	0.82	1.23	1.04	0.84	1.29	0.94	0.73	1.22
	3year	1.01	0.81	1.25	1.04	0.83	1.31	0.95	0.74	1.23
	1 year	0.99	0.80	1.23	1.02	0.81	1.28	0.93	0.72	1.20
Gallbladder	5 year	0.85	0.66	1.08	0.82	0.63	1.07	0.80	0.58	1.09
	3year	0.89	0.69	1.16	0.86	0.65	1.14	0.86	0.62	1.18
	1 year	0.96	0.74	1.25	0.91	0.69	1.21	0.93	0.68	1.27

Female genital	5 year	1.12	0.77	1.64	1.16	0.80	1.69	1.04	0.66	1.62
	3year	1.18	0.79	1.74	1.19	0.80	1.76	1.06	0.67	1.67
	1 year	1.14	0.76	1.70	1.15	0.77	1.71	1.05	0.67	1.65
Kidney	5 year	1.44	0.94	2.20	1.42	0.91	2.21	1.09	0.65	1.84
	3year	**1.58**	**1.02**	**2.45**	1.54	0.98	2.42	1.23	0.73	2.07
	1 year	**1.68**	**1.08**	**2.61**	**1.61**	**1.02**	**2.55**	1.32	0.78	2.21
Breast	5 year	1.07	0.67	1.72	1.06	0.66	1.71	1.33	0.75	2.36
	3year	1.09	0.67	1.78	1.07	0.66	1.76	0.80	0.45	1.43
	1 year	0.98	0.60	1.60	0.96	0.58	1.57	0.73	0.41	1.29
Prostate	5 year	0.78	0.53	1.17	0.81	0.50	1.30	0.86	0.49	1.50
	3year	0.86	0.57	1.30	0.91	0.56	1.48	1.01	0.57	1.76
	1 year	0.90	0.59	1.37	0.99	0.60	1.64	1.12	0.64	1.96
Non-Hodgkin’s	5 year	0.92	0.63	1.35	0.97	0.64	1.48	1.03	0.63	1.70
	3year	0.90	0.60	1.34	0.97	0.62	1.51	1.03	0.62	1.71
	1 year	0.88	0.59	1.32	0.90	0.58	1.42	0.93	0.56	1.54
Esophagus	5 year	0.88	0.60	1.27	1.04	0.69	1.57	1.22	0.72	2.06
	3year	0.96	0.65	1.42	1.14	0.74	1.74	1.36	0.81	2.30
	1 year	1.12	0.75	1.66	1.21	0.78	1.86	**1.99**	**1.19**	**3.31**
Leukemia	5 year	1.19	0.76	1.86	1.15	0.70	1.90	1.08	0.60	1.95
	3year	1.15	0.72	1.84	1.19	0.71	2.00	1.14	0.63	2.08
	1 year	1.20	0.75	1.91	1.29	0.77	2.17	1.26	0.70	2.27
Bladder	5 year	1.12	0.68	1.85	1.02	0.58	1.81	0.71	0.35	1.48
	3year	1.11	0.66	1.88	1.06	0.59	1.91	0.82	0.39	1.72
	1 year	0.99	0.58	1.70	0.91	0.49	1.69	0.70	0.33	1.48
Oral and Pharynx	5 year	0.98	0.61	1.56	1.14	0.69	1.90	1.00	0.55	1.80

	3year	0.96	0.59	1.57	1.13	0.66	1.91	1.00	0.55	1.80
	1 year	1.07	0.65	1.74	1.25	0.73	2.12	1.16	0.66	2.06
Brain	5 year	1.12	0.83	1.52	1.26	0.91	1.75	0.95	0.62	1.44
	3year	1.00	0.72	1.39	1.14	0.80	1.62	0.82	0.53	1.26
	1 year	1.00	0.72	1.37	1.12	0.80	1.58	0.84	0.55	1.26
Multiple myeloma	5 year	**1.42**	**1.15**	**1.75**	**1.51**	**1.20**	**1.89**	**1.65**	**1.22**	**2.24**
	3year	**1.53**	**1.23**	**1.90**	**1.65**	**1.30**	**2.10**	**1.80**	**1.33**	**2.44**
	1 year	**1.58**	**1.27**	**1.95**	**1.72**	**1.36**	**2.18**	**1.84**	**1.38**	**2.45**

*Primary model

[1]Model 1: PM + age + sex

[2]Model 2: Model 1 + Health insurance premium + Employment status+ Cigarette smoking status + Cigarette smoking amount (pack per day) + Cigarette smoking period (year) + Alcohol consumption + Physical activity + Nutrition + BMI + Family history of cancer

[3]Model 3: Model 2 + district-level of Elderly population, completeness of high school graduates, Gross Regional Domestic Product, and Population density + Area type + Health screening participation

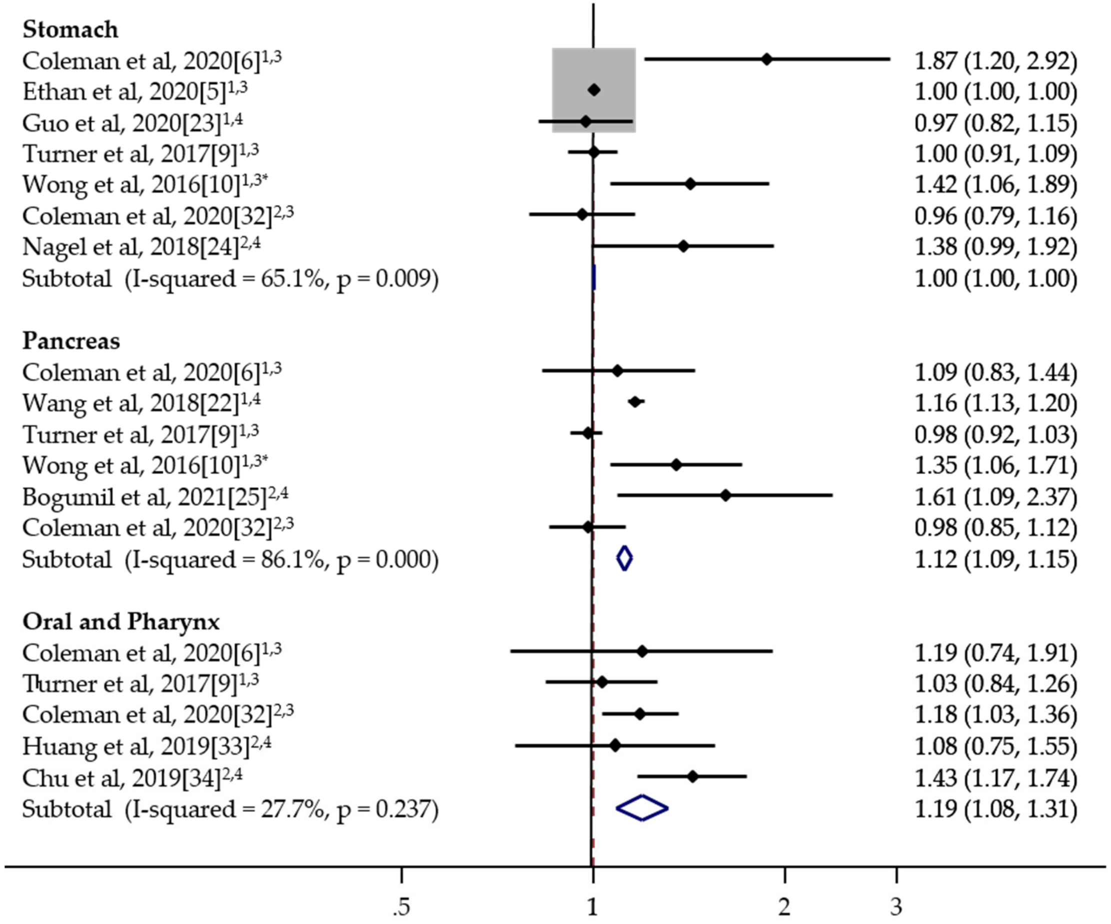

[1]Motality study

[2]Incidence study

[3]Study for comparison across cancers

[4]Study for individual cancer

*Wong et al, 2016 stomach cancer includes esophageal cancer as upper digestive tract; pancreas cancer includes liver cancer as accessory organs.

Figure S4. Hazard ratios and 95% confidence intervals of mortality or incidence of stomach, pancreas, and oral and pharynx cancer for long-term exposure to $PM_{2.5}$.

Table S7. Hazard ratios and 95% confidence intervals of lung, pancreas, non-Hodgkin's, esophagus, and oral and pharynx cancer mortality for a 10μg/m³ increase in individual-level $PM_{2.5}$ concentrations for the previous 5 years in the primary model (model 2) by definition of cancer deaths based on both single and multiple cancer sites versus single sites only in 87,608 National Health Insurance Service-National Sample Cohort subjects in the Seoul Metropolitan Area.

		Definition of cancer death							
		Single and multiple cancer sites				Single cancer site only			
Cancer type	Exposure period	Case	HRs	LL	UL	Case	HRs	LL	UL
Lung	5 year		1.55	0.73	3.26		3.03	0.85	10.78
	3year	367	1.62	0.79	3.31	273	2.13	0.91	5.03
	1 year		1.68	0.84	3.35		2.34	1.03	5.31
Pancreas	5 year		3.47	1.05	11.51		5.77	1.47	22.71
	3year	129	3.60	1.16	11.23	95	5.53	1.53	20.00
	1 year		2.47	0.81	7.59		3.03	0.85	10.78
Non-Hodgkin's	5 year		1.44	0.12	17.83		1.62	0.12	22.02
	3year	35	0.79	0.07	9.17	31	0.94	0.08	11.68
	1 year		0.57	0.06	5.76		0.67	0.06	7.09
Esophagus	5 year		2.43	0.21	27.82		2.71	0.21	35.45
	3year	35	2.87	0.27	30.72	33	2.95	0.25	35.17
	1 year		3.02	0.34	27.29		3.27	0.34	31.56
Oral and Pharynx	5 year		7.14	0.40	126.86		28.39	0.90	894.43
	3year	25	5.31	0.36	78.93	18	12.50	0.52	299.72
	1 year		8.14	0.80	83.10		9.99	0.61	162.99

Table S8. HRs and 95%CI of lung, pancreas, non-Hodgkin's, esophagus, and oral and pharynx cancer mortality for a 10μg/m^3 increase in individual-level PM2.5 concentrations for the previous 5, 3, and 1 years in primary model (model 2) for the comparison between including and excluding lung cancer patients before 2006 using National Health Insurance Service-National Sample Cohort subjects in the Seoul Metropolitan Area.

		Including cancer patients				Excluding cancer patents			
Cancer type	Exposure period	Case	HRs	LL	UL	Case	HRs	LL	UL
Lung	5 year		1.55	0.73	3.26		1.60	0.24	10.76
	3year	367	1.62	0.79	3.31	53	3.27	0.56	19.06
	1 year		1.68	0.84	3.35		2.81	0.50	15.82
Pancreas	5 year		3.47	1.05	11.51		2.49	0.30	20.74
	3year	129	3.60	1.16	11.23	46	4.26	0.60	30.16
	1 year		2.47	0.81	7.59		3.64	0.60	22.03
Non-Hodgkin's	5 year		1.44	0.12	17.83		0.62	0.02	22.97
	3year	35	0.79	0.07	9.17	17	0.33	0.01	11.50
	1 year		0.57	0.06	5.76		0.29	0.01	9.01
Esophagus	5 year		2.43	0.21	27.82		1.74	0.00	1958.95
	3year	35	2.87	0.27	30.72	5	0.91	0.00	1722.42
	1 year		3.02	0.34	27.29		0.42	0.00	267.05
Oral and Pharynx	5 year		7.14	0.40	126.86		0.44	0.00	208.71
	3year	25	5.31	0.36	78.93	7	0.15	0.00	51.70
	1 year		8.14	0.80	83.10		0.17	0.00	42.33
